# Dr. George B. Wheeler

**Published:** 1896-06

**Authors:** 


Dr. George B. Wheeler died at the Willard state hospital, April 7, 1896, as the result of appendicitis. He had been a medical interne in that institution since May 16, 1895, but had recently been appointed on the staff.

